# Laboratory and Numerical Investigation of Microwave Heating Properties of Asphalt Mixture

**DOI:** 10.3390/ma12010146

**Published:** 2019-01-04

**Authors:** Haopeng Wang, Yue Zhang, Yi Zhang, Shuyin Feng, Guoyang Lu, Lintao Cao

**Affiliations:** 1 Section of Pavement Engineering, Faculty of Civil Engineering and Geosciences, Delft University of Technology, Stevinweg 1, 2628 CN Delft, The Netherlands; 2 Institute of Highway Engineering, RWTH Aachen University, Mies-van-der-Rohe-Street 1, 52074 Aachen, Germany; 25yuezhang@web.de (Y.Z.); lu@isac.rwth-aachen.de (G.L.); 3 School of Highway, Chang’an University, Xi’an 710064, Shaanxi, China; yizhang@chd.edu.cn; 4 Department of Civil Engineering, University of Bristol, BS8 1TR Bristol, UK; shuyin.feng@bristol.ac.uk; 5 School of Civil Engineering and Architecture, Hubei University of Arts and Science, Xiangyang 441053, China

**Keywords:** asphalt mixture, microwave heating, steel slag, dielectric loss, electromagnetic, numerical simulation

## Abstract

Microwave heating is an encouraging heating technology for the maintenance, recycling, and deicing of asphalt pavement. To investigate the microwave heating properties of asphalt mixture, laboratory tests and numerical simulations were done and compared. Two types of Stone Mastic Asphalt (SMA) mixture samples (with basalt aggregates and steel slag aggregates) were heated using a microwave oven for different times. Numerical simulation models of microwave heating of asphalt mixture were developed with finite element software COMSOL Multiphysics. The main thermal and electromagnetic properties of asphalt mixture, served as the model input parameters, were measured through a series of laboratory tests. Both laboratory-measured and numerical simulated surface temperatures were recorded and analyzed. Results show that the replacement of basalt aggregates with steel slag aggregates can significantly increase the microwave heating efficiency of asphalt mixture. Numerical simulation results have a good correlation with laboratory test results. It is feasible to use the developed model coupling electromagnetic waves with heat transfer to simulate the microwave heating process of asphalt mixture.

## 1. Introduction

Microwave heating has been widely applied in various industrial fields, such as drying, material preparation, food processing, healthcare, etc. [1] Microwaves have the potential to provide rapid, uniform, high efficient, safe, and environment-friendly heating of materials [2]. Due to the above advantages of microwave heating, there have been increased interests in utilizing microwave heating in the asphalt paving industry. Specifically, three main applications in pavement engineering include asphalt pavement maintenance (such as crack healing, pothole patching, rut repair, etc.) [3,4,5], recycling of the old asphalt pavement (heating of reclaimed asphalt pavement using a microwave power unit) [6] and snow melting or deicing [7,8]. The main mechanism of microwave heating is the dielectric loss of a material under the microwave filed, including polarized relaxation loss and conductive loss [2]. Asphalt mixture usually consists of about 5% of asphalt, about 95% of coarse aggregate, fine aggregate and other mineral powders [9]. When asphalt mixture is exposed to microwave radiation, heat is generated through conversion of the energy of the electromagnetic field. In the conventional heating methods, such as hot-air heating and infrared heating, energy is transferred from the surfaces of the material internally by convection, conduction, and radiation [10]. In contrast, microwave heating is achieved by molecular excitation inside the material without relying on the temperature gradient. Therefore, microwave heating is a direct energy conversion process rather than heat transfer from external heat sources [3]. This fundamental difference in transferring energy endows microwave heating many exclusive advantages. More recently, induction heating was introduced in asphalt pavement. It is based on the Faraday’s electromagnetic induction theory and only applicable to the conductive asphalt materials [11,12]. However, the skin effect caused by the induced eddy currents causes high surface temperature and low internal temperature, thus producing a large temperature gradient through the system [13]. Therefore, microwave heating is a promising, competitive, and effective heating technology.

Nevertheless, the microwave heating efficiency of ordinary asphalt mixture is relatively low due to the low microwave absorbing properties. The capability of a material in absorbing microwave energy can be described by its dielectric properties [14]. The dielectric property of a material is usually expressed by the dielectric permittivity ε^*^ in Equation (1).

(1) $$\varepsilon^{*}\left(f\right)=\varepsilon \prime \left(f\right)-i\varepsilon \prime \prime \left(f\right)$$

where ε′ is the dielectric constant; ε″ is the dielectric loss factor; f is the frequency of the external electric field, and $i=\sqrt{-1}$. The dielectric property is highly dependent on the frequency. The dielectric constant determines the amount of storable energy in the material in the form of an electric field. The dielectric loss factor indicates how much of that energy can dissipate in the form of heat. The loss tangent tanδ, defined as ε″/ε′, reflects the material’s ability of transforming microwave energy into heat. The complex permittivity of asphalt mixtures are influenced not only by frequency and temperature, but also by other properties such as density, asphalt type and content, aggregate type and size, void ratio, and moisture content [15]. It was reported that asphalt has a very low loss tangent of about 0.001. Most of the conventional mineral aggregates, such as albite, marble, orthoclase, and quartz, have poor microwave absorbing characteristics [16]. Therefore, efforts were put into improving the microwave absorbing efficiency of asphalt mixture by adding microwave absorbers or using magnetite-bearing aggregates, such as taconite aggregate mineral and steel slag. Other attempts to improve microwave-absorbing capability included the addition of graphite, carbonyl iron powders (CIPs), carbon nanotubes, steel wool, and ferrite particles [10,13,17,18]. The magnetism of asphalt mixture introduced by ferrite additives is responsible for the magnetic loss during microwave heating. Permeability is the parameter to describe the degree of magnetization that a material experiences under the influence of an external magnetic field. Similar to the complex permittivity, the real part of permeability (μ′) is related to energy storage, and the imaginary part (μ″) implies the magnetic loss in particles [10]. Therefore, by improving the permittivity and permeability of asphalt mixture, the microwave heating properties will be enhanced.

Through various time and material consuming laboratory tests, it can be found microwave heating is a promising technology for asphalt pavement recycling and maintenance. However, fewer studies applied numerical modelling to investigate the microwave heating process and mechanism of asphaltic materials [10,13,19]. The aim of this study was to investigate the microwave heating properties of different types of asphalt mixtures through both laboratory test and numerical simulation.

## 2. Materials and Methods

### 2.1. Materials and Mix Design

In this study, the used asphalt binder was neat Pen-70 asphalt from Shell. Table 1 presents the basic properties of the binder. Basalt aggregates, steel slag aggregates and limestone fillers were used to produce asphalt mixture samples. Various properties of both aggregates are shown in Table 2. Polyester fiber was added as the drain-down stabilizer at the dosage of 0.3% by the total weight of the mix. As industrial waste, steel slag contains some metal oxides, especially transition metal such as ferric oxide. It was reported that steel slag can significantly influence the thermal and electromagnetic properties of asphalt mixture [10,20]. Steel slag was used as a partial substitute for basalt [21]. Due to the strong absorption of asphalt, fine steel slag aggregates were not chosen to substitute for fine basalt aggregates smaller than 2.36 mm. Since the specific gravity of steel slag is different from basalt aggregate, the equivalent-volume method was used to replace the coarse basalt aggregates above 2.36 mm with steel slag.

**Figure 1:**
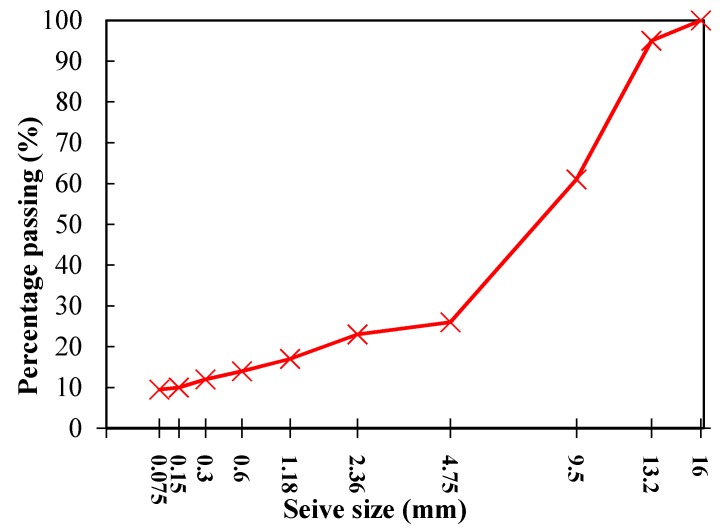
Mix gradation of Stone Mastic Asphalt-13 (SMA-13).

Stone Mastic Asphalt (SMA) with 13.2-mm nominal maximum aggregate size, designated as SMA-13, was used in this study. Gradation SMA-13 shown in Figure 1 was designed in accordance with the standard Marshall Design method (ASTM D6927) [22]. Two types of asphalt mixture samples, control mix with basalt aggregates (SMA-B) and mix with steel slag substitution (SMA-S), were prepared. To avoid the influence of varying grain sizes, both aggregates were sieved into different sieve sizes and then mixed into the specific gradation. The optimum asphalt content for SMA-B was 6.2%. The same asphalt content was chosen for SMA-S to minimize the control variable. The air void for both types of the mix was around 4.0%. Standard Marshall cylindrical specimens (101.6 mm in diameter and 63.5 mm in height) were fabricated for microwave heating test.

### 2.2. Thermal Properties Measurement

Thermal conductivity, thermal diffusivity and specific heat capacity are the three most important factors that affect the microwave heating process of materials, which refers to heat transfer phenomena [23]. The thermal conductivity was measured through a steady-state method using a heat flow meter (HFM 446, NETZSCH Group, Selb, Germany) according to ASTM C518 [24]. A slab specimen (15 cm × 15 cm × 4 cm) was placed between two plates with temperature gradient, and the heat flow created by the well-defined temperature difference is measured with a heat flux sensor. In this case, 5 °C and 35 °C were set as the constant temperatures for the cold plate and the hot plate respectively. The thermal conductivity can be calculated based on the acquired data according to the Fourier’s Law for heat conduction (Equation (2)).

(2) q=−k∇T

where q is the conductive heat flux; k is the thermal conductivity; T is the transient temperature. The specific heat capacity $C_{p}$ was also measured by the heat flow meter. With the total heat consumption required to heat the sample and temperature development, the specific heat capacity can be determined at a certain temperature. Thermal diffusivity (α) is the coefficient that characterizes the rate of heat energy diffusion throughout a material when it is exposed to a fluctuating thermal environment. Thermal diffusivity is calculated as thermal conductivity divided by density (ρ) and specific heat capacity at a constant pressure.

(3) $$\alpha =\frac{k}{\rho \times c_{p}}$$

### 2.3. Electromagnetic Properties Measurement

Electromagnetic parameters, including complex permittivity and complex permeability, are the main indicators to quantify the microwave absorbing efficiency of a material. To obtain these above parameters of asphalt mixture specimens, measurements were carried out with an Agilent E5071C vector network analyzer (Santa Clara, CA, USA) using the free-space method [25]. The detailed measurement system and calculation process can be found in Reference [22]. The electrical conductivity of asphalt mixture was measured by the simple two-probe method [9,26].

### 2.4. Temperature Measurement under Microwave Heating

**Figure 2:**
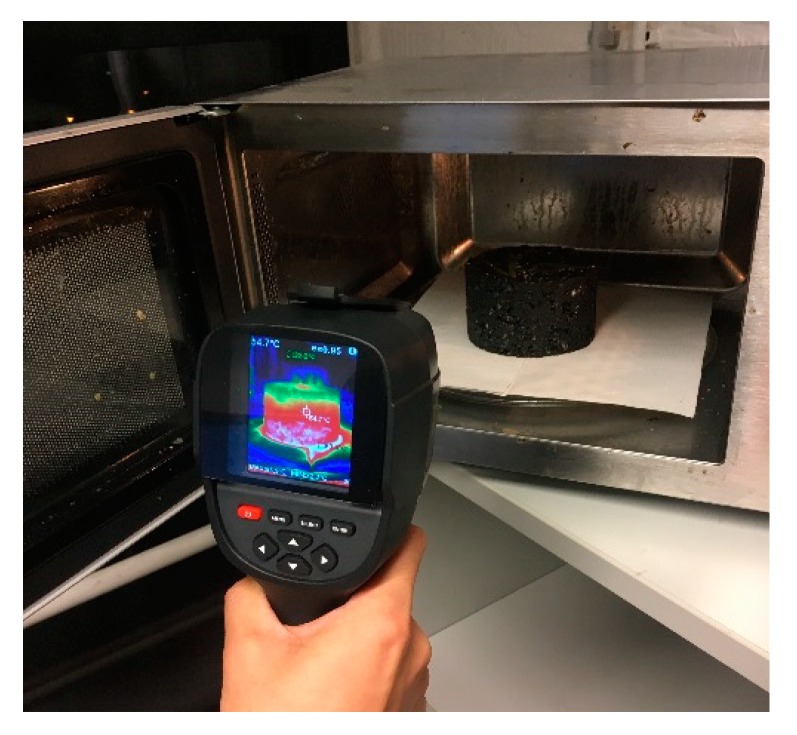
Surface temperature measurement of the specimen in the microwave oven.

Asphalt mixture samples were heated using a commercial microwave oven (Galanz P100M25ASL-H4, Guangdong Galanz Enterprise Co, ltd., Foshan, China) with an input of 1200 W and a 220 V, 50 Hz power supply. The oven can generate microwaves of up to 1000 W at an excitation frequency of 2.45 GHz, which corresponds to a wavelength of 122.4 mm. Each type of asphalt mixture sample has two replicates due to the potential variation of test results. The cylindrical specimen (Φ101.6 mm × 63.5 mm) was placed on the center of the glass plate in the microwave oven. The surface temperature was measured every 20 s by swiftly opening the door using a thermal infrared camera as shown in Figure 2 [19]. The total heating time was 120 s. The average temperature value of six randomly selected points from the specimen surface was calculated as the experimental temperature.

## 3. Numerical Simulation

As discussed before, microwave heating is a multiphysics phenomenon that involves the physics of electromagnetic waves and heat transfer. The rapidly varying electric and magnetic fields lead to four sources of heating. First, any electric field applied to a conductive material will generate eddy currents. In addition, a time-varying electric field will cause dipolar molecules within the material to oscillate back and forth to generate molecular friction. A time-varying magnetic field applied to a conductive material will also induce current flow. For certain types of magnetic materials, the hysteresis losses also make contribution to the heating [27]. To simulate the electro-magneto-thermal phenomenon in a real-time mode, the finite element software COMSOL Multiphysics (Version 5.3, COMSOL BV, Zoetermeer, The Netherlands) has been utilized for modelling microwave heating of asphalt mixture.

### 3.1. Electromagnetic Waves

Electromagnetic analysis of asphalt mixture on a macroscopic level involves solving Maxwell’s equations subject to certain boundary conditions. These equations can be formulated in partial differential form, which can be handled by the finite element method.

(4a) $$\nabla \times H=J+\frac{\partial D}{\partial t}$$

(4b) $$\nabla \times E=-\frac{\partial B}{\partial t}$$

(4c) $$\nabla \cdot D=\rho_{e}$$

(4d) ∇·B=0

To apply the Maxwell equations self-consistently, the constitutive relations describing the macroscopic behaviors of matter under the influence of fields need to be obtained. Assuming asphalt mixture is an isotropic and linear material, the constitutive equations can be formulated as follows.

(5a) J=σE

(5b) D=εE

(5c) B=μH

where H is the magnetic field intensity; J is the electric current density; D is the electric displacement or electric flux density; E is the electric field intensity; B is the magnetic flux density; $\rho_{e}$ is the electric charge density; σ is the material electrical conductivity; ε is the material permittivity; and μ is the material permeability.

### 3.2. Heat Transfer

Applied microwave energy is transformed into power based on the electromagnetic field distribution at a particular location. The absorbed power term is considered a source term in heat transfer equations to calculate transient temperature profile. The diffusion of heat into continua is governed by:

(6) $$\rho C_{p}\frac{\partial T}{\partial t}=\nabla \cdot \left(k\nabla T\right)+Q_{e}$$

where ρ is the density; $C_{p}$ is the specific heat at constant pressure; k is the thermal conductivity; T is the temperature at time t; and $Q_{e}$ is the internal heat source (absorbed power). The surface of the matter exchanges heat with surrounding air by convection expressed as:

(7) $$-n\cdot q=h\left(T-T_{a}\right)$$

where q is the conductive heat flux, which is proportional to the temperature gradient in Equation (2); h is the surface convective coefficient; n is the normal vector on the boundary; T is the transient temperature; and $T_{a}$ is the ambient temperature.

### 3.3. Multiphysics Coupling

**Figure 3:**
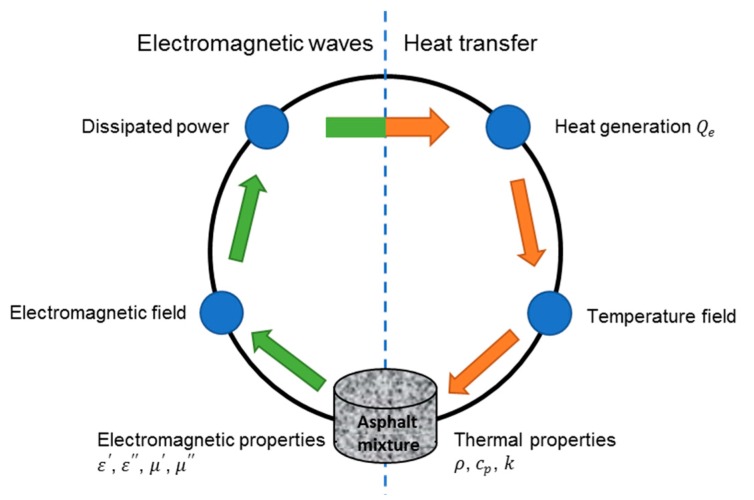
Schematic flow chart of coupling electromagnetic and thermal fields [28].

The electro-magneto-thermal phenomenon often encountered in microwave heating is usually solved in a coupled manner because the power dissipation calculated from electromagnetic fields influences other physical phenomenon, such as heat transfer, component evaporation, and microstructural change in heated materials. These complex physical situations result in rapid changes in material properties, which in turn makes the problem highly nonlinear [27]. However, the nonlinearity in this study was not considered because of the difficulty to measure the input material parameters of such an in homogenous material. The process of coupling electromagnetic waves and heat transfer in microwave heating is shown in Figure 3. The distributed heat source, which includes resistive heating (ohmic heating) and magnetic losses in Equation (8) [28], is computed from a stationary electromagnetic analysis in the frequency domain. Then a transient heat transfer simulation showing how the heat redistributes in the asphalt mixture samples was followed. In the software, the frequency domain study is only used for the electromagnetics interface, whereas the time-dependent study is only applicable to the heat transfer interface. Notice that the electromagnetic heat source will be computed first, and then used in the time-dependent heat transfer study step.

(8a) $$Q_{e}=Q_{rh}+Q_{ml}$$

(8b) Qrh=12Re(J·E)

(8c) Qml=12Re(iωB·H)

where $Q_{rh}$ is the resistive heating of dielectric material due to the electric current; $Q_{ml}$ is the magnetic loss of magnetic material interacting with the magnetic field component of microwave. Re() is the real part of the variable.

### 3.4. Model Definition

**Figure 4:**
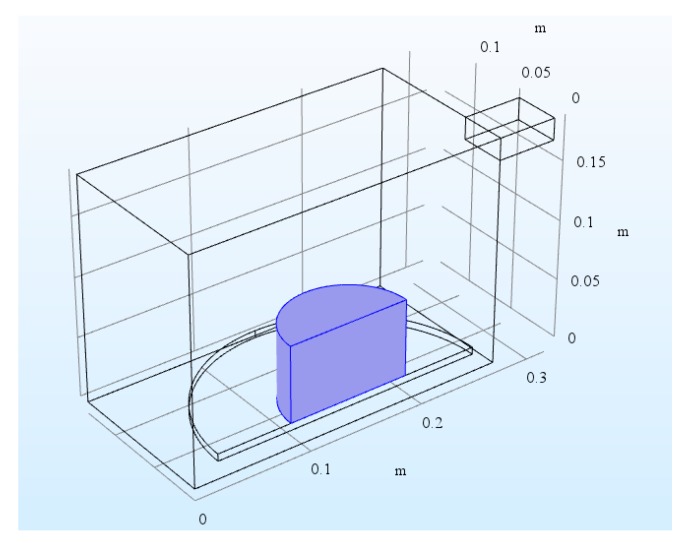
Geometry of microwave oven, asphalt mixture sample, and waveguide feed.

**Figure 5:**
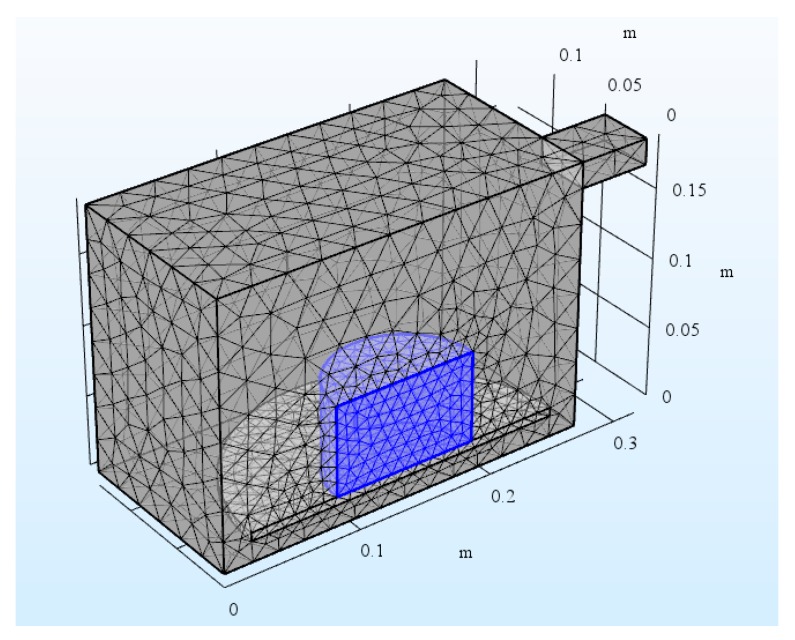
Mesh of microwave oven, asphalt mixture sample, and waveguide feed.

The microwave oven is a metallic box connected to a 2.45 GHz microwave source via a rectangular waveguide. The dimensions of the oven are 267 mm (width) × 270 mm (depth) × 188 mm (height). The size of the waveguide is 50 mm (width) × 78 mm (depth) × 18 mm (height). There is a cylindrical glass plate near the bottom of the oven. A cylindrical asphalt mixture sample was placed on top of the glass plate. The microwave operates at 1000 W, but because the symmetrical model was built to reduce the model size by one half, only 500 W was input in the simulation. The symmetry cut is applied vertically through the oven, waveguide, asphalt mixture sample, and plate. The symmetrical geometry and 3D mesh are shown in Figure 4 and Figure 5, respectively. Copper was applied for the walls of the oven and waveguide in this model. The applied impedance boundary condition on these walls ensures the small resistive metals losses get accounted for. The symmetry cut has mirror symmetry for the electric field and is represented by the boundary condition as shown in Equation (9).

(9) n×H=0

where n is the outward unit normal vector to the port boundary; H is the magnetic field vector.

The rectangular port is excited by a transverse electric (TE) wave, which is a wave that has no electric field component in the propagating direction. At an excitation frequency of 2.45 GHz, TE_10_ mode is the only mode of propagation through the rectangular waveguide. The propagation constant β required for the port mode settings is frequency (v) dependent:

(10) $$\beta =\frac{2\pi }{c}\sqrt{v^{2}-v_{c}^{2}}$$

where c is the speed of light and $v_{c}$ is the cutoff frequency.

### 3.5. Material Properties

As discussed before, to implement the finite element model of microwave heating on asphalt mixtures, their material properties need to be obtained as the input parameters. Specifically, the thermal and electromagnetic parameters of both SMA-B and SMA-S mixtures were presented in Table 3 and Table 4. The presented data were the averaged values from test results of three replicates. It can be noted that the replacement of basalt with steel slag decreased the thermal conductivity and specific heat capacity of asphalt mixture, while the thermal diffusivity was increased. Steel slag has a porous inter-structure. Many small pores within the porous steel slag obstruct the heat transfer process, which is accounted for the decrease of the thermal conductivity of asphalt mixture. The high porosity of steel slag also contributes to the heat retention characteristics, which is responsible for the decrease of heat capacity [29]. In terms of electromagnetic properties, SMA-S has higher electrical conductivity than SMA-B. The addition of steel slag also increases both permittivity and permeability of asphalt mixture as shown in Table 4. The improvement of the electromagnetic properties of SMA-S is due to the ferric components and other metal elements in steel slag.

## 4. Results and Discussions

### 4.1. Numerical Simulation Results

#### 4.1.1. Microwave Heat Source Distribution

**Figure 6:**
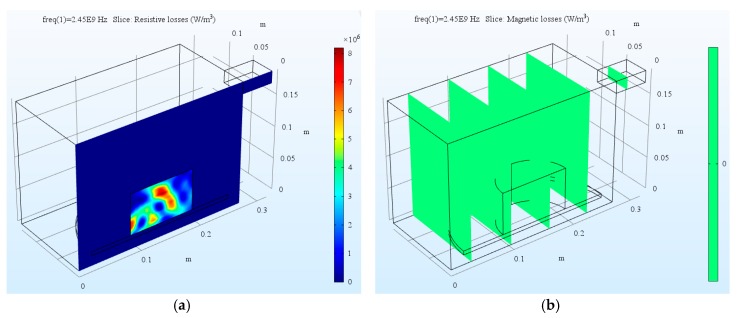
The dissipated microwave power distribution of asphalt mixture SMA-B: (**a**) Resistive loss; (**b**) Magnetic loss.

**Figure 7:**
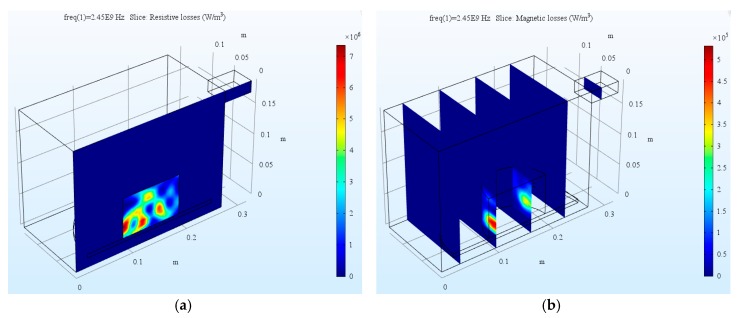
The dissipated microwave power distribution of asphalt mixture SMA-S: (**a**) Resistive loss; (**b**) Magnetic loss.

The numerical analysis using the current model took several minutes with common personal computer configuration. The distributed microwave heat source as a slice plot through the center of asphalt mixture sample SMA-B and SMA-S are shown in Figure 6 and Figure 7, respectively. It indicates that the resistive loss distribution shows a complicated oscillating pattern, which has several strong peaks inside the sample. Since sample SMA-B is a non-magnetic material, there is no magnetic loss during the microwave heating process. On the contrary, sample SMA-S has both resistive loss and magnetic loss. Through a volume integration of the microwave heating, the amount of resistive loss and magnetic loss, as well as the total power loss during the heating process were calculated in Table 5. The total microwave energy absorbed by the asphalt mixtures is more than 90% of the input microwave power (500 W). It is interesting to note that the resistive loss of SMA-S after microwave heating is lower than that of SMA-B. However, from the total heat source, SMA-S with steel slag aggregates has a higher microwave absorbing efficiency than SMA-B with basalt aggregates.

#### 4.1.2. Temperature Distribution of Test Samples

**Figure 8:**
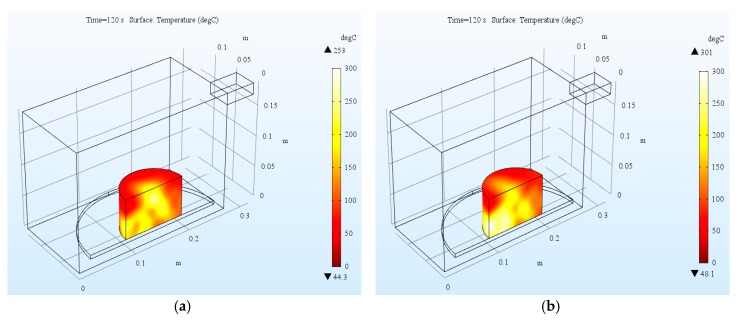
Surface temperature distribution of asphalt mixture: (**a**) SMA-B; (**b**) SMA-S.

The temperature distribution of two types of asphalt mixture after 120 s simulative microwave heating are presented in Figure 8. It looks like the surface temperature of SMA-S is higher than that of SMA-B from the temperature contour plot. Quantitative analysis will be conducted in the following part. It should be emphasized that the rectangular cross section of the cylindrical sample seen as the surface area is actually the internal part of the sample due to the symmetric treatment of the model. It is obvious that the temperature distribution of the asphalt mixture specimen during heating was not uniform. The internal temperatures were higher the surface ones. This is possibly due to the fact that heat dissipation on the surface of a specimen is greater than its interior. This simulation results coincide with the laboratory results [5,24,30]. When heating the asphalt mixture to certain temperatures, the inside water contents start boiling and transporting heat as steam to outer layers. Asphalt may start flowing due to softening, resulting in the change of air voids and skeleton structure. These physio-chemical changes of the mix constituents also affect the electromagnetic properties of the asphalt mixture. The simple microwave absorption and heat conduction model used here does not capture these nonlinear effects. However, the model can serve as a starting point for a more advanced analysis.

### 4.2. Comparison between Laboratory and Simulation Results

**Figure 9:**
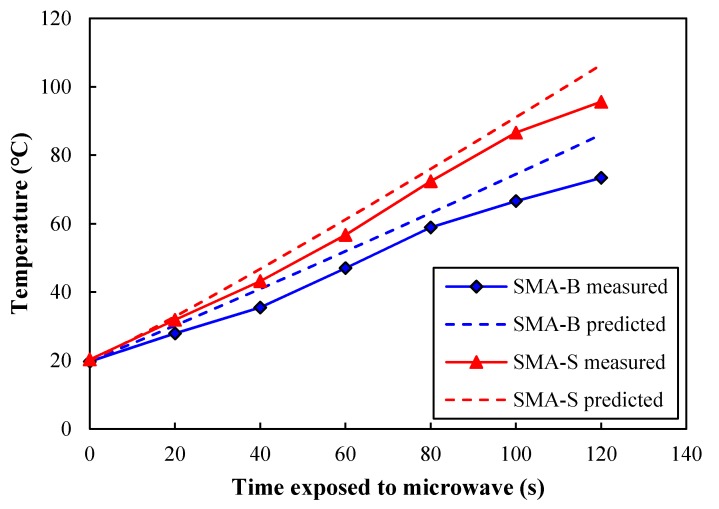
Surface temperature comparison of asphalt mixture between laboratory test and numerical simulation.

The lateral surface temperatures of laboratory test and numerical simulation are compared in Figure 9. Here the simulative surface temperature values were averaged through area integral. It is obvious that numerical simulation results have a good correlation with the experimental results for both types of asphalt mixtures. However, the numerically simulated temperatures are somewhat higher than the laboratory test results. This is possibly due to the temperature loss during the laboratory measurement for several seconds. In addition, the nonlinear effects during the heating process can be the reason for this, which needs to be further included in the model. Nevertheless, it is feasible to use a numerical method to simulate the microwave heating process of asphalt mixture.

More precisely, the initial temperatures, final temperatures and heating rates of asphalt mixture samples during microwave heating are summarized in Table 6. SMA-S has a better microwave heating performance than SMA-B. The final lateral surface temperature of SMA-S reached 95.6 °C while that of SMA-B was only 73.4 °C. The higher heating rate of SMA-S than SMA-B confirms the higher microwave absorbing efficiency of SMA-S due to the addition of steel slag.

## 5. Conclusions

This study investigated the microwave heating properties of two types of asphalt mixture through both laboratory test and numerical simulation. The main thermal and electromagnetic properties of used asphalt mixtures were explored through laboratory tests. The following conclusions can be drawn based on the study:

(1) The partial replacement of basalt aggregates with steel slag aggregates improve the electromagnetic properties of asphalt mixture. Microwave heating of asphalt mixture sample containing steel slag includes both resistive heating and magnetic heating due to the altered permeability of the sample.
(2) Asphalt mixture sample containing steel slag aggregates has a higher microwave heating efficiency than ordinary asphalt mixture sample with basalt aggregates.
(3) There is a good correlation between laboratory measured temperatures and numerically simulated temperatures of asphalt mixture samples.
(4) It is feasible to use the developed FEM model, which coupled electromagnetic waves with heat transfer, to simulate the microwave heating process of asphalt mixture.

For further research, the size effect of test samples and specific material parameters, such as moisture content, air voids, asphalt content, aggregate properties, etc. should be considered. Developing more advanced numerical models which consider the nonlinear effects and time discretization will be a challenge. In addition, the microstructural changes and mechanical performance of asphalt mixtures after microwave heating will be further investigated.

## Figures and Tables

**Table 1:** Basic properties of asphalt binder.

Properties	Value
Penetration (25 °C, 100 g, 5 s, 0.1 mm)	71
Ductility (5 cm/min, 5 °C, cm)	32.2
Softening point (R&B, °C)	47.5
Flash point (°C)	272
Rotational viscosity (60 °C, Pa·s)	203
Wax content (%)	1.6
Density (15 °C, g/cm^3^)	1.032

**Table 2:** Basic properties of aggregates.

Aggregate	Specific Gravity (g/cm^3^)	Water Absorption (%)	Crushing Value (%)	Asphalt Affinity (%)	Abrasion Loss (%) (Los Angeles)
Basalt	2.82	0.72	12.8	>85	14.6
Steel slag	3.47	1.26	12.2	>95	13.8

**Table 3:** Thermal parameters of asphalt mixtures.

Mixture Type	Density (kg/m^3^)	Thermal Conductivity (W/(m·K))	Specific Heat Capacity (J/(kg·K))	Thermal Diffusivity (m^2^/s)
SMA-B	2530	1.508	918.5	6.49 × 10^−7^
SMA-S	2632	1.446	756.5	7.26 × 10^−7^

**Table 4:** Electromagnetic parameters of asphalt mixtures at 2.45 GHz.

Mixture Type	σ	$\varepsilon^{\prime }$	$\varepsilon^{\prime \prime }$	$\mu^{\prime }$	$\mu^{\prime \prime }$
SMA-B	4.26 × 10^−9^	5.34	0.49	1.0	0
SMA-S	3.85 × 10^−7^	5.68	0.52	1.03	0.006

**Table 5:** The distributed heat source of asphalt mixtures at 2.45 GHz.

Mixture Type	Resistive Losses (W)	Magnetic Losses (W)	Total Heat Source (W)
SMA-B	455.19	0	455.19
SMA-S	442.06	28.37	470.43

**Table 6:** Microwave heating performance of asphalt mixture samples on the lateral surface.

Mixture Type		Initial Temperature (°C)	Final Temperature (°C)	Heating Rate (°C/s)
SMA-B	Experiment	19.7	73.4	0.448
	Simulation	20.0	86.1	0.551
SMA-S	Experiment	20.3	95.6	0.623
	Simulation	20.0	106.5	0.721
